# Supplementary material for: An Engineered Protein-Based Building Block (Albumin Methacryloyl) for Fabrication of a 3D In Vitro Cryogel Model
Source: Gels. 2022 Jun 25;8(7):404. doi: 10.3390/gels8070404 (PMC9324498; doi:10.3390/gels8070404)
Supplement: Supplementary file 1 [file gels-08-00404-s001.zip › gels-1756998-supplementary_v1.pdf]

gels

Article

An Engineered Protein-Based Building Block (Albumin Methacryloyl) for Fabrication of a 3D In Vitro Cryogel Model

Xueming Niu [1], Mian Lin [1] and Bae Hoon Lee [1,2,*]

[1] Wenzhou Institute, University of Chinese Academy of Sciences, Wenzhou 325011, China; xueming.niu@uq.edu.au (X.N.); linmian@wiucas.ac.cn (M.L.)
[2] Oujiang Laboratory (Zhejiang Lab for Rengerative Medicine, Vision and Brain Health), Wenzhou 325000, China
* Correspondence: bhlee@ucas.ac.cn

Supplementary Materials

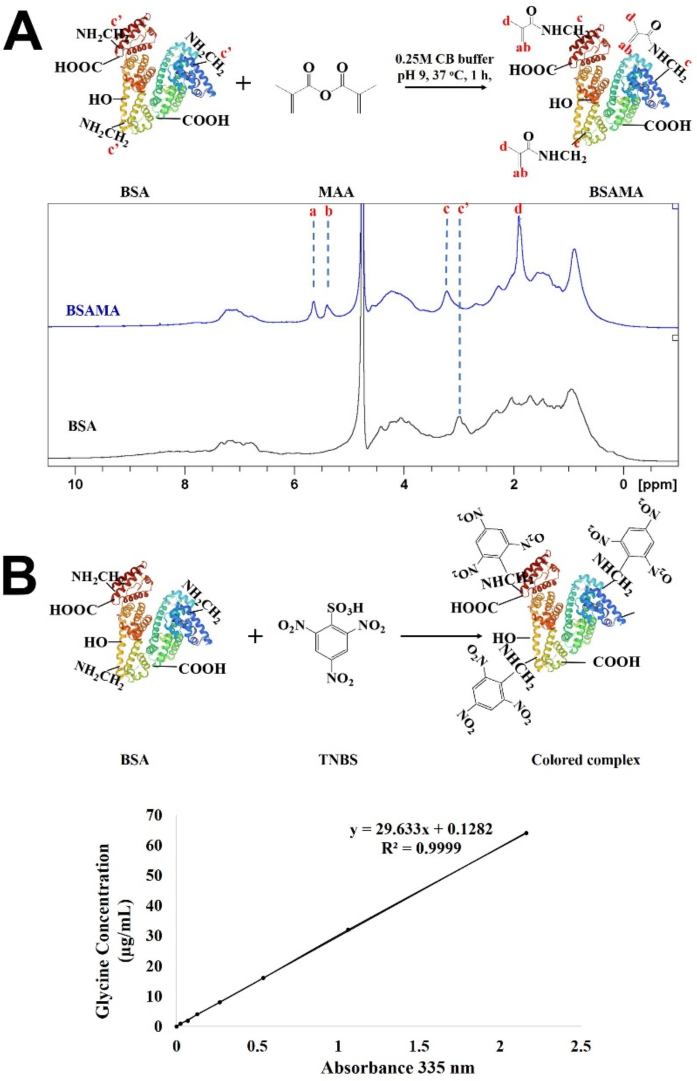

Figure S1. (A) Schematic illustration of the reaction between BSA and MAA. BSA, bovine serum albumin; MAA, methacrylic anhydride; CB, carbonate-bicarbonate; BSA-MA, BSA methacryloyl. ^{1}H-NMR spectra of BSA and BSA-MA samples with a 93.09% degree of methacryloylation. (a, b)

Acrylic protons (2H) of methacrylamide groups (at 5.4 and 5.6 ppm). (c) Methylene protons (2H) of reacted lysine groups at around 3.2 ppm are shifted from those (c′) of unreacted lysine groups at around 3.0 ppm. (d) Methyl protons (3H) of methacrylamide groups at around 1.9 ppm. Quantification of the degree of methacryloylation (DM) in BSAMA using TNBS reagent. **(B)** Scheme of the TNBS reaction; the standard curve was used to determine the molar concentration of the free amino groups.

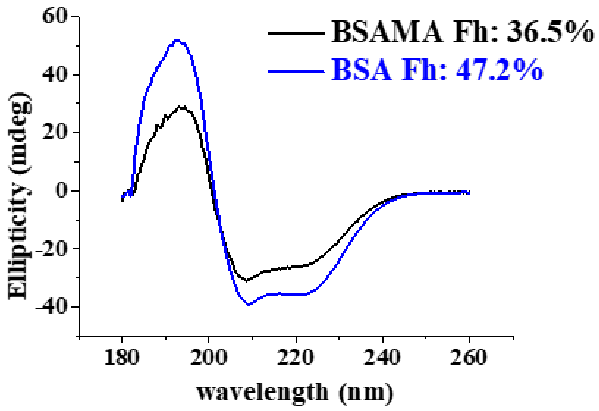

Figure S2. CD spectra of BSA and BSA-MA in DI H_2O. Fractional helicity percentages of BSA and BSAMA are 47.2% and 35.6%, respectively.

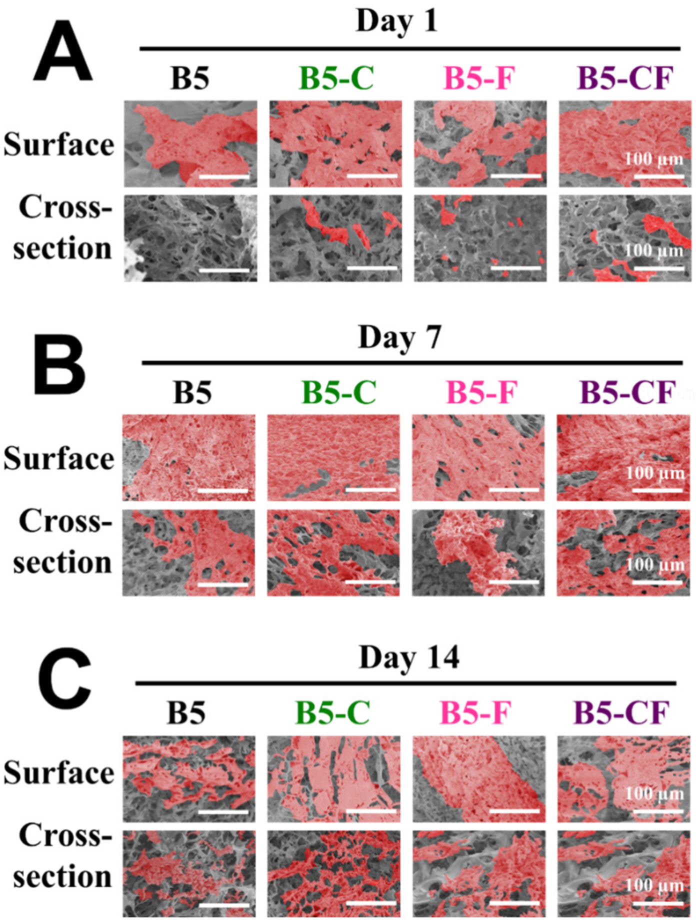

Figure S3. Effect of ECM-coated BSAMA cryogels on in vitro cellular migration at day 1, day 7, and day 14. Cell distribution in cryogels was investigated by SEM images of the surface and inside of

the scaffolds (with cells colored in red). **(A)** Cell distribution at day 1, **(B)** day 7, and **(C)** day 14. Scale bar: 100 μm.

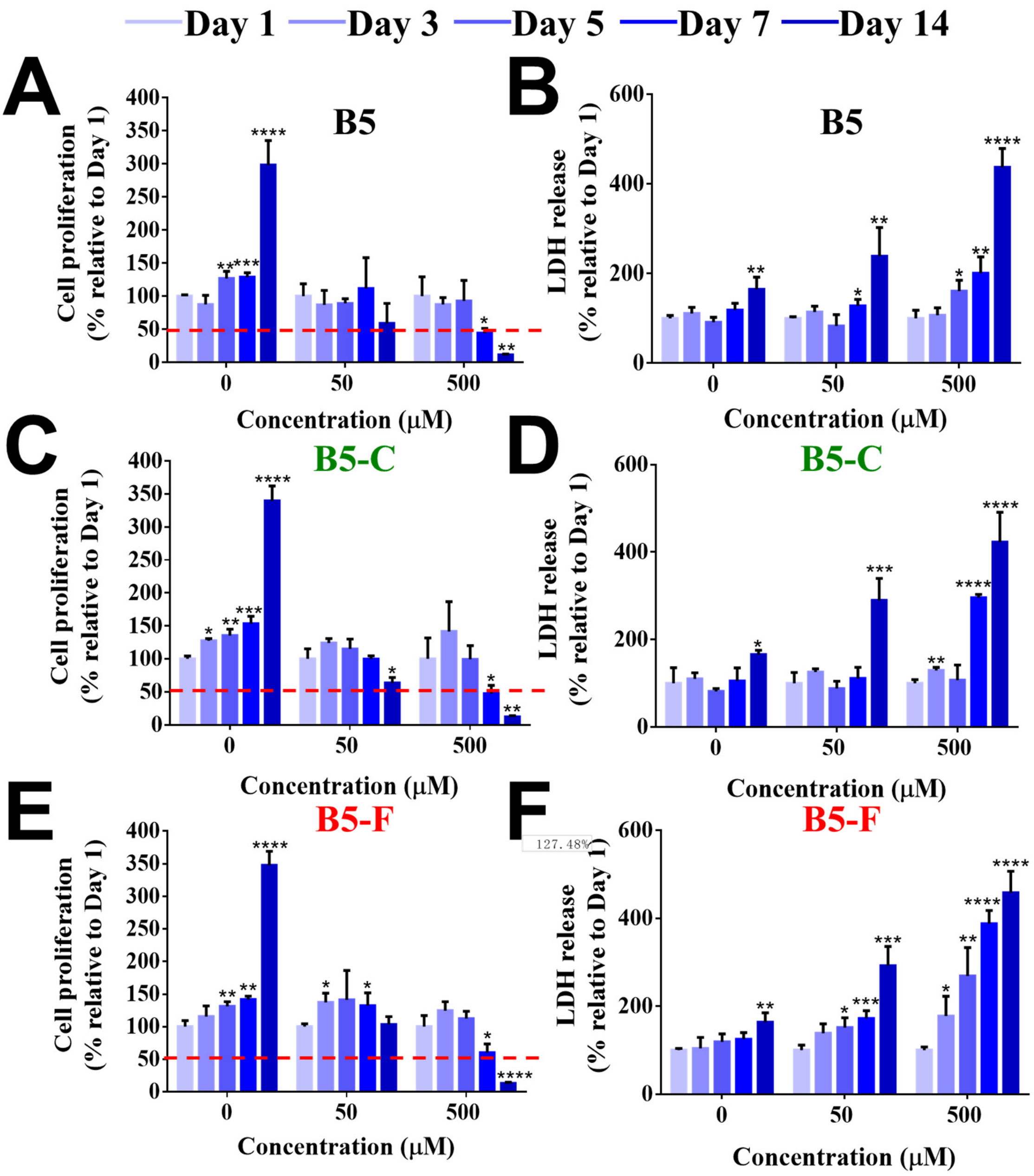

Figure S4. Evaluation of FIAU-induced cytotoxicity in engineered albumin-based scaffolds at day 1, day 3, day 5, day 7, and day 14. Cell proliferation and cytotoxicity (LDH release) of B5 (**A**, **B**); B5-C (**C**, **D**); B5-F (**E**, **F**) at day1, day 3, day 5, day 7, and day 14 after FIAU treatment (n = 3, *: P < 0.05, **: P < 0.01, ***: P < 0.005, ****: P < 0.001), compared to the same cryogel type at day 1. The red-dashed lines indicate the 50% relative cell proliferation.

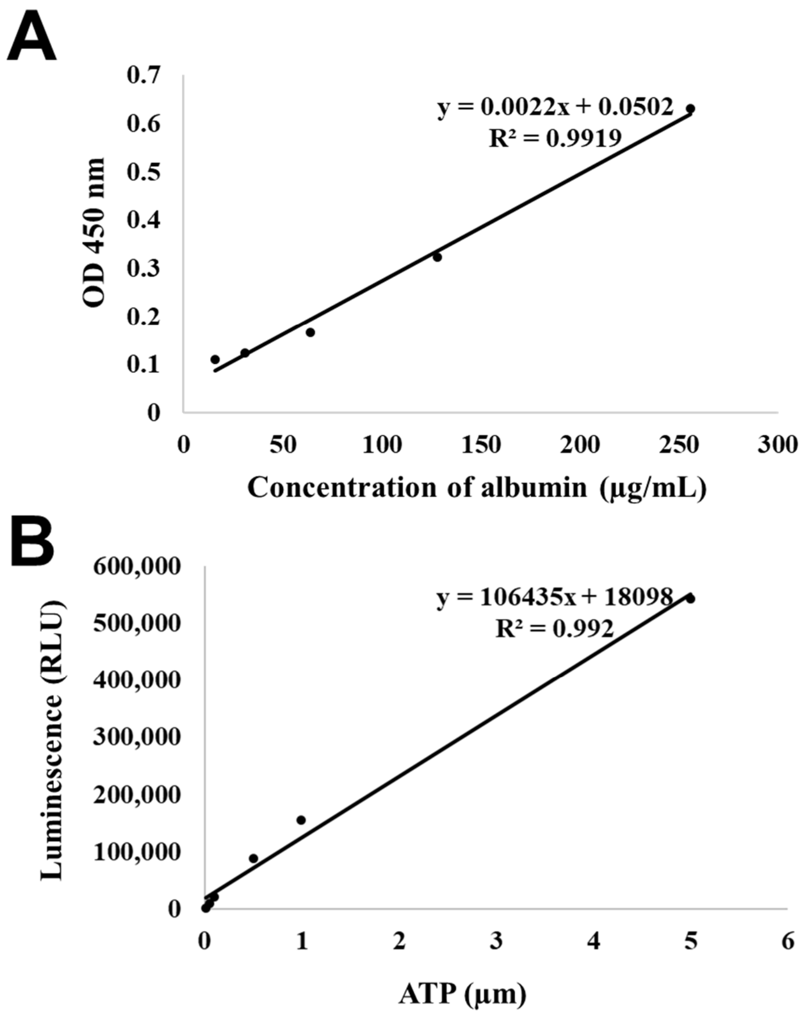

Figure S5. (A) Standard curve of human serum albumin measured by ELISA. **(B)** Standard curve of ATP measured by ELISA.

Table S1. The preparation of albumin-based cryogels with three different concentrations.

	5% BSAMA (B5)	10% BSAMA (B10)	15% BSAMA (B15)
APS (15 mg mL^{-1})	0.900	0.600	0.495
TEMED	0.155	0.078	0.062

Note: the unit is % w/v; APS: ammonium persulfate; TEMED: N,N,N,N-tetramethylethylenediamine. To avoid fast polymerization, a lower amount of APS and TEMED was used in a higher concentration of BSAMA.

Table S2. List of the primer sequences used in amplification.

Target genes	Forward (5′-3′)	Reverse (5′-3′)
**AAT**	**ATGCTGCCCAGAAGACAGATA**	**CTGAAGGCGAACTCAGCCA**
**Albumin**	**TGCAACTCTTCGTGAAACCTATG**	**ACATCAACCTCTGGTCTCACC**
**CYP3A4**	**AAGTCGCCTCGAAGATACACA**	**AAGGAGAGAACACTGCTCGTG**
**CYP3A7**	**AAACTTGGCCGTGGAAACCT**	**CCTTACGGAAGGACAAAGCATTT**
**G6Pase**	**GTGTCCGTGATCGCAGACC**	**GACGAGGTTGAGCCAGTCTC**
**HNF4α**	**CACGGGCAAACACTACGGT**	**TTGACCTTCGAGTGCTGATCC**
**HNF6**	**GAACATGGGAAGGATAGAGGCA**	**GTAGAGTTCGACGCTGGACAT**
**E-cadherin**	**CGAGAGCTACACGTTCACGG**	**GGGTGTCGAGGGAAAAATAGG**
**N-cadherin**	**TCAGGCGTCTGTAGAGGCTT**	**ATGCACATCCTTCGATAAGACTG**
**Claudin-1**	**CCTCCTGGGAGTGATAGCAAT**	**GGCAACTAAAATAGCCAGACCT**
**ZO-1**	**CAACATACAGTGACGCTTCACA**	**CACTATTGACGTTTCCCCACTC**
**GAPDH**	**CCATGGGGAAGGTGAAGGTC**	**CTCGCTCCTGGAAGATGGTG**
