# Supplementary material for: An Engineered Protein-Based Building Block (Albumin Methacryloyl) for Fabrication of a 3D In Vitro Cryogel Model
Source: Gels. 2022 Jun 25;8(7):404. doi: 10.3390/gels8070404 (PMC9324498; doi:10.3390/gels8070404)
Supplement: Supplementary file 1 [file gels-08-00404-s001.zip › Video S1 and S2_v2.pptx]

## Slide 1
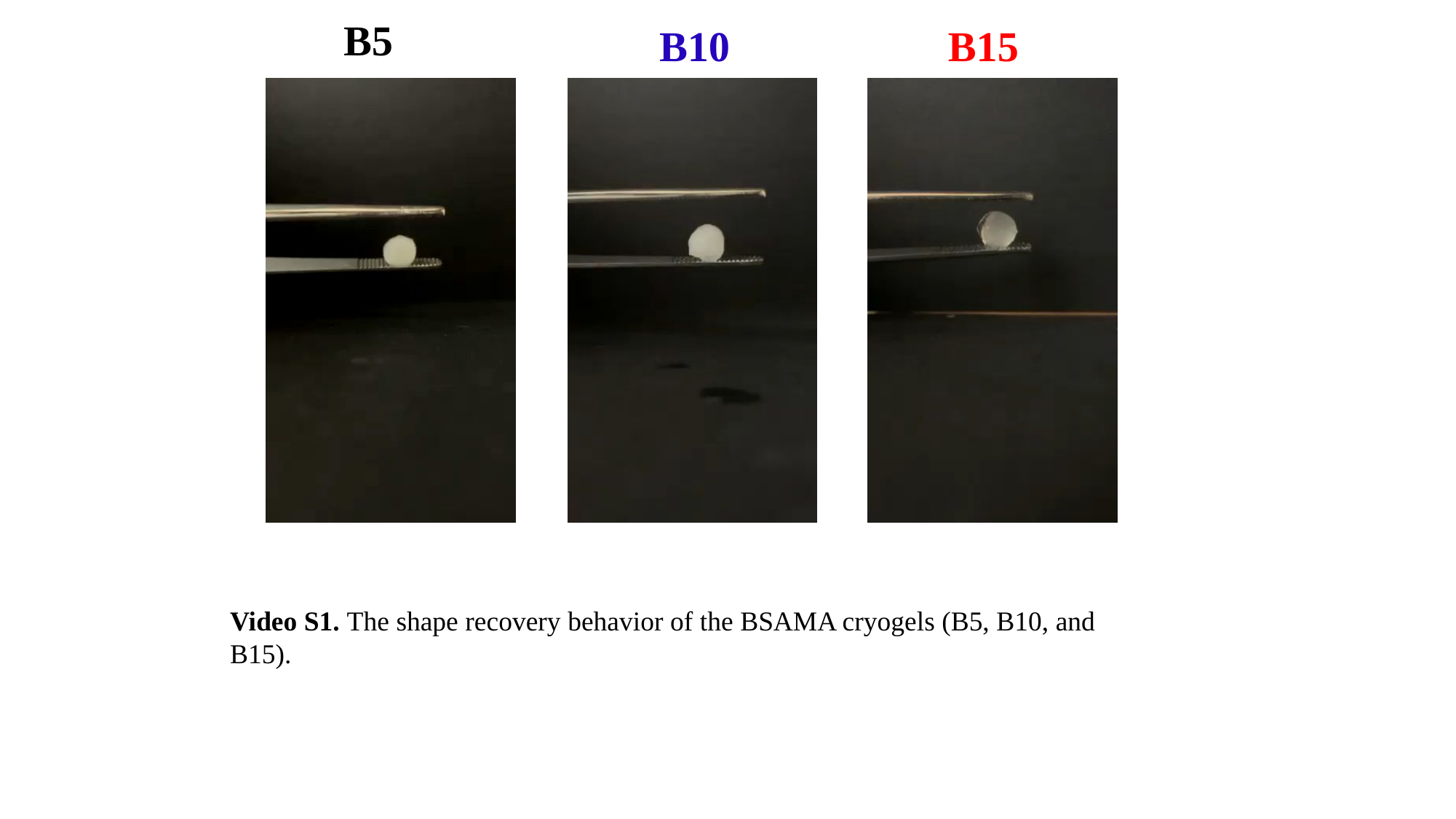

B5
B10
B15
Video S1. The shape recovery behavior of the BSAMA cryogels (B5, B10, and B15).

## Slide 2
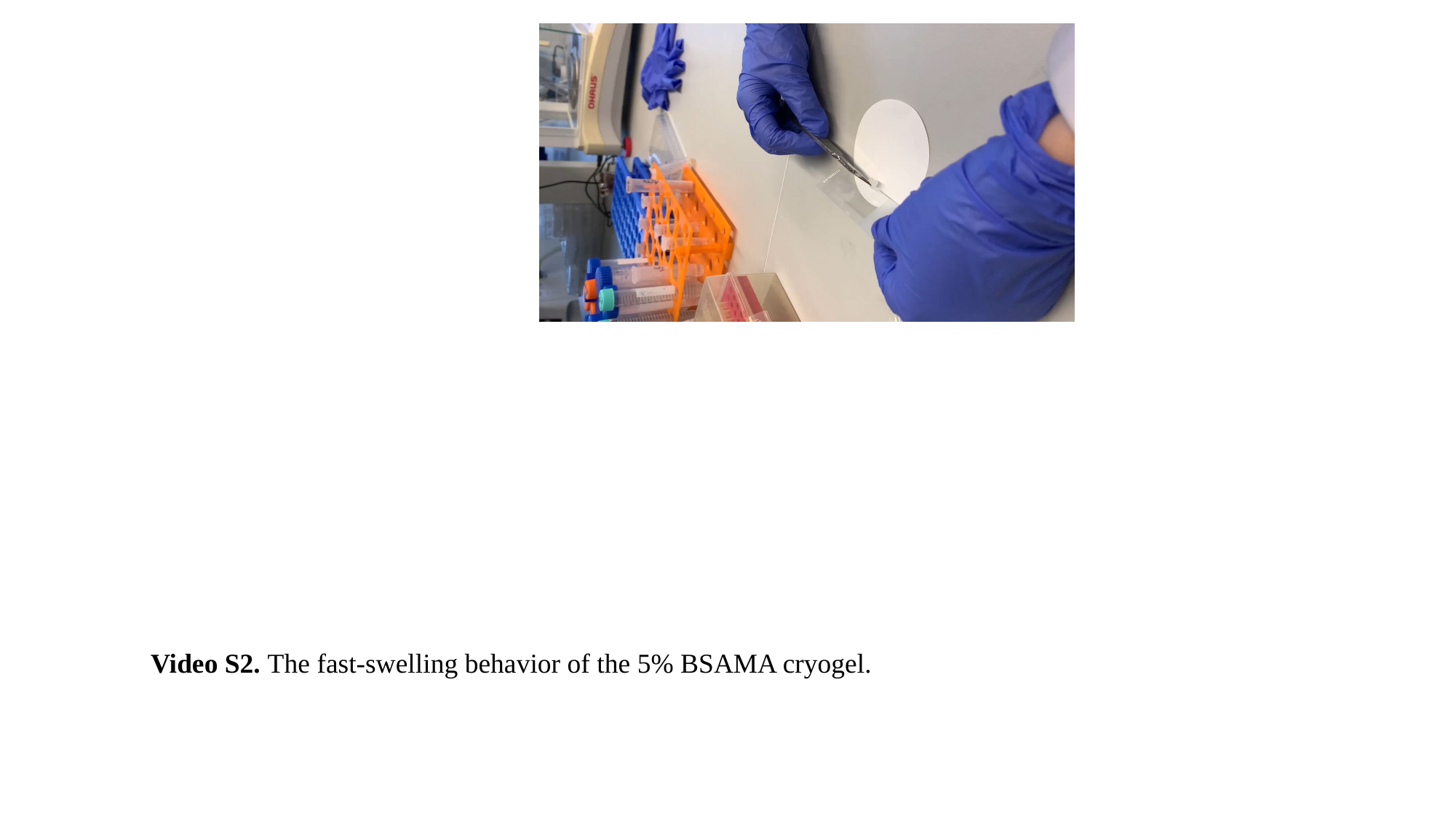

Video S2. The fast-swelling behavior of the 5% BSAMA cryogel.
